# Effects of Sevoflurane and Propofol on Organ Blood Flow in Left Ventricular Assist Devices in Pigs

**DOI:** 10.1155/2015/898373

**Published:** 2015-10-25

**Authors:** Paloma Morillas-Sendín, Emilio Delgado-Baeza, María Jesús Delgado-Martos, Mónica Barranco, Juan Francisco del Cañizo, Manuel Ruíz, Begoña Quintana-Villamandos

**Affiliations:** ^1^Department of Anesthesiology and Intensive Care, Gregorio Marañón University General Hospital, 28007 Madrid, Spain; Department of Experimental Medicine and Surgery, Gregorio Marañón University General Hospital, 28007 Madrid, Spain ^2^ Department of Experimental Medicine and Surgery, Gregorio Marañón University General Hospital, 28007 Madrid, Spain; ^3^Department of Cardiac Surgery, Gregorio Marañón University General Hospital, 28007 Madrid, Spain; ^4^Department of Pharmacology, Faculty of Medicine, Complutense University, 28040 Madrid, Spain

## Abstract

The aim of this study was to assess the effect of sevoflurane and propofol on organ blood flow in a porcine model with a left ventricular assist device (LVAD). Ten healthy minipigs were divided into 2 groups (5 per group) according to the anesthetic received (sevoflurane or propofol). A Biomedicus centrifugal pump was implanted. Organ blood flow (measured using colored microspheres), markers of tissue injury, and hemodynamic parameters were assessed at baseline (pump off) and after 30 minutes of partial support. Blood flow was significantly higher in the brain (both frontal lobes), heart (both ventricles), and liver after 30 minutes in the sevoflurane group, although no significant differences were recorded for the lung, kidney, or ileum. Serum levels of alanine aminotransferase and total bilirubin were significantly higher after 30 minutes in the propofol group, although no significant differences were detected between the groups for other parameters of liver function, kidney function, or lactic acid levels. The hemodynamic parameters were similar in both groups. We demonstrated that, compared with propofol, sevoflurane increases blood flow in the brain, liver, and heart after implantation of an LVAD under conditions of partial support.

## 1. Introduction

Ventricular assist devices (VADs) are a promising therapeutic option for patients with advanced heart failure. VADs can act as a bridge to transplantation, as a destination therapy for patients with contraindications to transplantation, or as a bridge to a future recovery [1–3]. In the last few decades, VADs have been increasingly used in patients with end-stage heart failure, because heart transplantation is limited by a marked lack of donors [4].

The main purpose of a VAD is to maintain perfusion of vital organs. To improve the clinical output of the VAD, it is necessary to optimize perioperative conditions (continuous-flow VAD, hemodynamic monitors, and anesthetic drugs) [5, 6]. Although several studies show the effects of the VAD on organ blood flow (heart, brain, liver, and kidney) [7–9], the effect of anesthetics on organ blood flow in patients with a VAD has not been analyzed to date. Several studies have reported data on the response of organ blood flow to the administration of various anesthetics [10–13], although this effect remains unclear for VADs.

Given the beneficial effects of volatile anesthetics (sevoflurane) compared with intravenous anesthesia (propofol) on organ blood flow during cardiovascular surgery [14–17], we hypothesized that, compared with propofol, sevoflurane would increase organ blood flow in patients with a left VAD (LVAD). The aim of this study was to investigate differences between the effect of sevoflurane-based volatile anesthetic and that of propofol-based intravenous anesthetics on organ blood flow (brain, liver, heart, kidney, lung, and intestine) and to assess markers of tissue injury after implantation of an LVAD (continuous centrifugal pump) under conditions of partial support in a porcine model.

## 2. Methods

The animals used in our experiment were from the farm of the Technological Institute of Agrarian Development (EX 013-C) (Community of Madrid, Spain). The pigs were moved from this farm to the Experimental Medicine and Surgery Unit, Gregorio Marañón University General Hospital (ES280790000087), where they remained under a controlled environment until the intervention (20–22°C and relative humidity of 55%). The study was performed in accordance with European Union guidelines on the protection of animals used for experimental and other scientific purposes (Directive 2010/63/EU and Spanish Royal Decree RD 53/2013 BOE) and was approved by the Ethics Committee, Gregorio Marañón University General Hospital, Madrid, Spain.

### 2.1. Experimental Design

The study was conducted with ten healthy minipigs. Animals were block-randomized (Microsoft Excel 2003) to receive either propofol in continuous perfusion as anesthetic maintenance (propofol group, *n* = 5) or sevoflurane (sevoflurane group, *n* = 5).

#### 2.1.1. Anesthesia Protocol

The animals were simultaneously premedicated with intramuscular ketamine 20 mg/kg (Ketolar, Parke-Davis, Madrid, Spain) and atropine 0.04 mg/kg (Atropina Braun, Serra-Pamies, Reus, Spain). Pulse oximetry and electrocardiographic monitoring were performed in the operating room. The pigs were provided with oxygen 100% via a face mask, a 20 G cannula was inserted into an ear vein, and anesthesia was induced with intravenous fentanyl 2.5 *μ*g/kg (Fentanest, Kern Pharma, Barcelona, Spain) and propofol 4 mg/kg (Diprivan 1%, AstraZeneca, Madrid, Spain). After intubation, the animal was connected to a volume-controlled ventilator (Dräger SA1, Dräger Medical AG, Lübeck, Germany) with FIO_2_ of 1, an inspiratory : expiratory ratio of 1 : 2, a tidal volume of 12–15 mL/kg, and the respiratory rate adjusted to maintain normocapnia as previously described [18]. Anesthesia was maintained with intravenous fentanyl (2.5 *μ*g/kg/30 min) in all animals and propofol in continuous infusion (11-12 mg/kg/h) (propofol group) or 2% sevoflurane (sevoflurane group). All animals received an infusion of saline solution (8 mL/kg/h). A 9 F arterial catheter was inserted into the right femoral artery and a pulmonary artery catheter (7.5 F Swan-Ganz CCOmbo catheter, Edwards Lifesciences, Irvine, CA, USA) connected to an oximetry monitor (Vigilance, Edwards Critical-Care Division, Irvine, CA, USA) was inserted into the right internal jugular vein.

#### 2.1.2. Surgical Protocol

**Figure 1:**
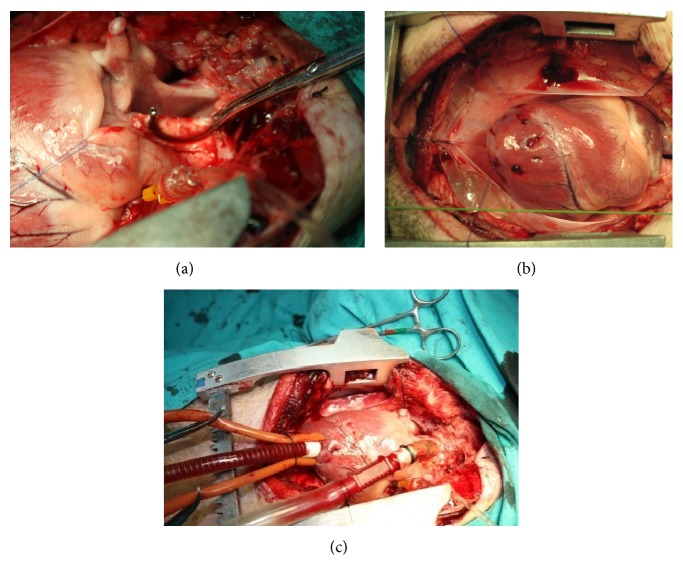
LVAD placement. Aortic partial cross-clamp (a). Implant of the input cannula through the apex of the left ventricle (b and c).

A Biomedicus 540 centrifugal pump was implanted in the minipigs undergoing continuous-flow support. After median sternotomy, the animal was heparinized at a dose of 4 mg/kg. An aortic partial cross-clamp was applied (just for anastomosing the output cannula of the LVAD to the aorta) and a 2 cm aortotomy performed (Figure 1(a)). The output cannula of the LVAD was anastomosed to the ascending aorta, and the input cannula (23 F Medtronic Ultraflex, Metdtronic Inc., Minneapolis, USA) was placed through the apex of the left ventricle. The implant of the input cannula is practiced by placing two circular sutures (Figure 1(b)), and then the cannula was placed with two turnstiles around the cannula (Figure 1(c)). Finally, both cannulas were connected to the device. LVAD placement was without cardiopulmonary bypass and without cardioplegia. Console parameters were adjusted to obtain a pump flow of 50% (partial support) of the baseline cardiac output (cardiac output before LVAD is initiated) using the pulmonary artery catheter for 30 minutes. Input flow was measured using an ultrasound transducer (EMTEC, Germany) attached to the input cannula of the device.

### 2.2. Organ Blood Flow Measurements

Colored microspheres (Dye-Trak, Triton Technology Inc., San Diego, CA, USA) were used to measure organ blood flow. Once the LVAD was implanted (before the start of LVAD, baseline), yellow microspheres (diameter of 12 microns) were injected into the left atrium (1.5 million microspheres per injection). The LVAD was then initiated, and violet microspheres were injected after 30 minutes of partial support. After each experiment, the animal was sacrificed using potassium chloride, and tissue samples of both brain hemispheres (right and left frontal lobe), heart (right and left ventricles), liver, lung (middle lobe of right lung), kidney, and ileum were obtained to measure organ blood flow. The basic principle of all deposition techniques for regional flow measurement is that the deposition is proportional to the flow (per unit volume or mass of tissue). Due to the movement of microspheres out of the capillaries into the interstitium, retention of microspheres is excellent. The idea is that deposited markers give a measure of flow per unit volume of tissue at the level of the capillaries. The microspheres were isolated from tissue by digestion with potassium hydroxide, they were centrifugated, the dyes were extracted from the colored microspheres, and the separation of colors and measurement of their concentration was performed by spectrometry [19, 20].

### 2.3. Markers of Tissue Injury

Serum levels of total bilirubin, alanine aminotransferase, aspartate aminotransferase, gamma-glutamyl transpeptidase, and alkaline phosphatase were evaluated as parameters of hepatobiliary function. Creatinine and urea were studied as parameters of renal function. Lactate dehydrogenase and lactate were measured as nonspecific indicators of tissue injury. All previously described markers of tissue injury and nitric oxide (NO) were studied at baseline (after implantation before turning it on) and 30 minutes after implantation of the LVAD.

### 2.4. Hemodynamic Measurements

The hemodynamic data included heart rate, mean arterial pressure, mean pulmonary arterial pressure, central venous pressure, pulmonary capillary wedge pressure, systemic vascular resistance index, pulmonary vascular resistance index, continuous cardiac output, and mixed venous oxygen saturation, all of which were recorded at baseline and 30 minutes after implantation of the LVAD. Body temperature was also studied.

### 2.5. Hematologic Parameters and Arterial Blood Gas Measurements

A femoral arterial catheter was used to perform the hematologic and blood gas analyses at baseline and 30 minutes after implantation of the LVAD.

### 2.6. Data Analysis and Statistics

The primary endpoint was organ blood flow in the LVAD, which was compared between the two groups. The variable was expressed as mean ± SEM. We used the Kolmogorov-Smirnov test to analyze the distribution of quantitative variables; between-group comparisons were based on the *t*-test for independent samples. Statistical significance was set at a *P* value of <0.05. The statistical analysis was performed using IBM SPSS Statistics for Windows, version 20.0 (IBM Corp, Armonk, NY, USA) and S-PLUS 6.1.

## 3. Results

### 3.1. Physiological Parameters

No differences were detected between the groups (sevoflurane versus propofol) in terms of age (143 ± 7 versus 126 ± 10 days, *P* = 0.28), weight (34 ± 1 versus 25 ± 3 kg, *P* = 0.052), or height (93 ± 2 versus 87 ± 1 cm, *P* = 0.07).

### 3.2. Effect of Anesthetics on Organ Blood Flow

**Figure 2:**
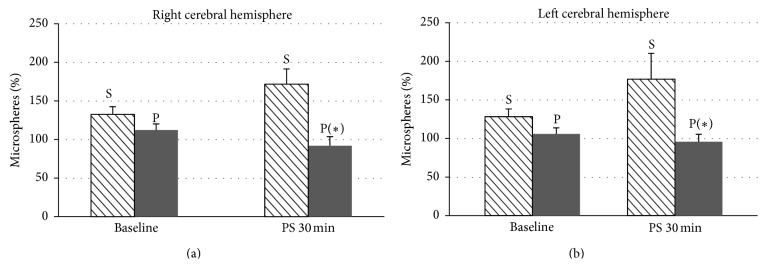
Date are expressed as the mean ± standard error of the mean. Cerebral blood flow in the right frontal lobe (a) and left frontal lobe (b) of pigs with a ventricular assist device in both groups, sevoflurane (S) and propofol (P), at baseline and after 30 minutes of partial support. Statistically significant differences are shown: ^*∗*^ *P* < 0.05 versus sevoflurane.

**Figure 3:**
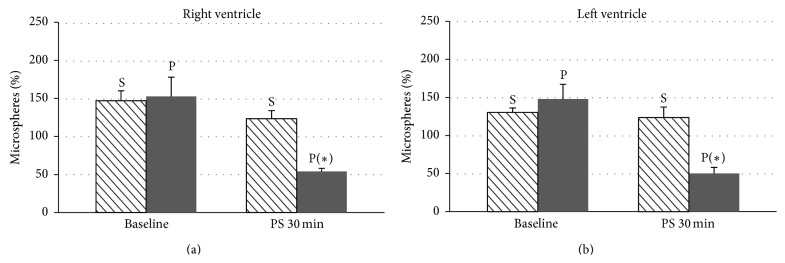
Date are expressed as the mean ± standard error of the mean. Blood flow in the right ventricle (a) and left ventricle (b) of pigs with a ventricular assist device in both groups, sevoflurane (S) and propofol (P), at baseline and after 30 minutes of partial support. Statistically significant differences are shown: ^*∗*^ *P* < 0.05 versus sevoflurane.

**Figure 4:**
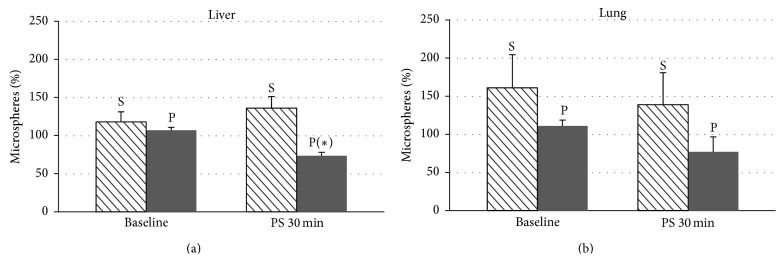
Date are expressed as the mean ± standard error of the mean. Blood flow in the liver (a) and lung (b) of pigs with a ventricular assist device in both groups, sevoflurane (S) and propofol (P), at baseline and after 30 minutes of partial support. Statistically significant differences are shown: ^*∗*^ *P* < 0.05 versus sevoflurane.

**Figure 5:**
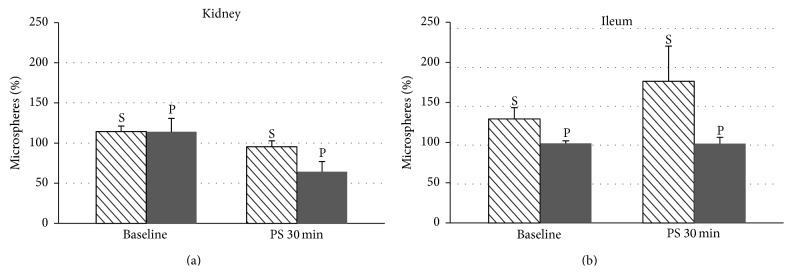
Date are expressed as the mean ± standard error of the mean. Blood flow in the kidney (a) and ileum (b) of pigs with a ventricular assist device in both groups, sevoflurane (S) and propofol (P), at baseline and after 30 minutes of partial support.

Blood flow was significantly higher in the brain (both frontal lobes) (Figures 2(a) and 2(b)), heart (both ventricles) (Figures 3(a) and 3(b)), and liver (Figure 4(a)) after 30 minutes of partial support in the sevoflurane group than in the propofol group, although no significant differences were recorded for the lung (Figure 4(b)), kidney (Figure 5(a)), or ileum (Figure 5(b)).

### 3.3. Effect of Anesthetics on Markers of Tissue Injury and Nitric Oxide

Serum levels of alanine aminotransferase and total bilirubin were significantly higher after 30 minutes of partial support in the group that received propofol. However, there were no significant differences between the groups in other parameters of liver function and kidney function or in lactic acid levels (Table 1). There were no differences between the groups in nitric oxide in plasma (Table 1).

### 3.4. Hemodynamic Parameters

No differences were found between the groups in pump flow of LVAD (propofol group 0.94 ± 0.09 L/min versus sevoflurane group 1.01 ± 0.09 L/min).

The hemodynamic parameters showed marked stability in both groups; there were no significant differences in either the sevoflurane group or the propofol group before implantation of the LVAD and after 30 minutes of partial support (Table 2).

### 3.5. Hematologic Parameters and Blood Gas Analysis

No statistically significant differences were found between the groups for hemoglobin and hematocrit after 30 minutes (Table 3). Arterial oxygenation, systemic arterial PCO_2_, bicarbonate, and pH were similar in both groups before implantation and after 30 minutes of partial support (Table 3).

## 4. Discussion

The results obtained show that, compared with propofol, anesthesia with sevoflurane increases blood flow in the brain, liver, and heart tissue after implantation of an LVAD under conditions of partial support in a porcine model. In addition, increased levels of serum markers of cellular injury in LVAD were observed with propofol. To our knowledge, this is the first study to demonstrate a beneficial effect of sevoflurane compared with propofol on organ blood flow in a Biomedicus 540 centrifugal pump in a porcine model. These findings justify further investigation to determine whether sevoflurane modifies organ blood flow in clinical settings.

The number of patients diagnosed with advanced heart failure is increasing worldwide, and LVAD is a pivotal treatment option for end-stage heart failure [21]. Because complications in the use of LVAD (multiple organ failure, right ventricular failure, neurological dysfunction, and arrhythmias) have been reported [22, 23], anesthesia and perioperative management of these critically compromised patients requires extensive monitoring, special anesthetic management with appropriate drugs, and expert postoperative care [24, 25].

### 4.1. Effect of Anesthetics on Organ Blood Flow

Several studies have reported changes in organ blood flow in response to the administration of volatile anesthetics and propofol [11–13, 26–28], although this effect has not been analyzed during implantation of an LVAD. Sevoflurane and propofol are frequently used as maintenance anesthetics during placement of an LVAD [29]. Some authors have associated reduced cerebral blood flow with both drugs [12]; however, we only found greater cerebral blood flow in sevoflurane-anesthetized animals with an LVAD. Patients with LVAD are associated with neurologic events. The most common causes are thromboembolism and hemorrhagic stroke and less frequent causes are ischemia due to low perfusion and air embolism [30]. However, we are not sure that a higher flow reduces the occurrence of ischemia due to air embolism. According to our results, sevoflurane could be a good option to lower the incidence of ischemia due to low perfusion in LVAD-supported patients.

The results of some studies support cardiac and hepatic protective effects of sevoflurane with respect to propofol after coronary artery surgery in humans [14, 16]. Our results also support the beneficial effect of sevoflurane compared with propofol on the heart and liver in LVAD. However, no differences were observed with sevoflurane compared with propofol for blood flow in other organs (lung, kidney, and intestine). The different blood flow response to sevoflurane could be explained by its dose-dependent effect [26–28].

Propofol and sevoflurane are used during cardiac surgery. Propofol exerts cardioprotective effects by different mechanisms: in the isolated heart, it attenuates metabolic changes induced by exogenously applied hydrogen peroxide [31], reduces infarct size by inhibition of GSK-3*β* activity (propofol induces cardiac preconditioning) [32], and attenuates ischemia-reperfusion injury mediated through increase in nitric oxide synthase activity and NO production (cardiac function and coronary flow are improved with propofol) [33, 34]. In our study there were no differences in NO between both groups: sevoflurane and propofol. Propofol attenuates the changes in myocardial tissue levels of adenine nucleotides and lactate during ischemia, reduces troponin I release on reperfusion after cardioplegic arrest in cardiopulmonary bypass in a model porcine [35], and shows antiarrhythmic effect during myocardial ischemia in rats [36]. However, cardiopulmonary bypass (CPB) is known to alter the plasma propofol concentrations (hemodilution, hypotension, hypothermia, isolation of the lungs from the circulation, and possible sequestration of drugs in the bypass circuit affect drugs plasma concentrations) [37].

Sevoflurane also induces preconditioning and attenuates myocardial ischemia/reperfusion injury via caveolin-3-dependent cyclooxygenase-2 inhibition, AMP-activated protein kinase, and antioxidative effects in experimental studies [38–40]. Clinical studies show that sevoflurane provides cardioprotection in patients undergoing coronary artery bypass graft (CABG) [41], and there is some data that shows that troponin T levels after off-pump CABG were lower in patients receiving sevoflurane compared to propofol [42]. In this context, cardioprotection by sevoflurane compared to propofol could also be superior in patients undergoing noncardiac surgery [43]. However, troponin T increased in patients undergoing repair of congenital heart defect with cardiopulmonary bypass anesthetized with propofol and sevoflurane [44]. In our study we did not use cardiopulmonary bypass (there was no ischemia/reperfusion) in LVAD implantation.

It is known that sevoflurane tends to cause vasodilatation cerebral, increases cerebral blood flow (CBF), and decreases cerebrovascular resistance [45]. However, propofol produces cerebral vasoconstriction indirectly by reducing cerebral metabolism and causes a decrease in CBF that is well matched to cerebral metabolism [46]. Regarding why in our study sevoflurane increases CBF, Kaisti et al. [12] confirmed that CBF is lower with propofol than with sevoflurane.

### 4.2. Effect of Anesthetics on Markers of Tissue Injury

The objective of a VAD is to maintain adequate organ perfusion [2]. However, liver dysfunction has been observed despite adequate hemodynamic support with an LVAD [47]. Some authors have reported hyperbilirubinemia in patients following implantation of an LVAD by hepatic sinusoid endothelial dysfunction [48] or cardiac congestion [49]. In our study, total bilirubin was higher in propofol-anesthetized animals than in sevoflurane-anesthetized animals; this finding was consistent with reduced blood flow in the liver and heart with respect to sevoflurane-anesthetized pigs.

Bernard et al. [50] found a portal blood flow decreased at both 1.2 and 2 MAC sevoflurane, whereas an increase in hepatic arterial blood flow was recorded at 2 MAC. These findings could explain why sevoflurane increases hepatic blood flow in our study.

### 4.3. Benefit of the Results for the Clinics

In our study, the use of sevoflurane leads to better outcomes after LVAD implantation by optimizing blood flow in the heart, brain, and liver. Although the necessary time to place an LVAD is short, the use of volatile anesthetic in cardiac surgery potentially reduces long-term cardiovascular complications and mortality [51]. Furthermore, intraoperative and postoperative sevoflurane administration in patients undergoing off-pump CABG could improve the cardioprotective effect compared with patients who received sevoflurane only in the intraoperative period [42]. It is possible because there is a disposable delivery system (AnaConDa) that is designed for halogenated sedation of patients in ICU [42]. LVAD, biventricular assist device (BIVAD), and extracorporeal membrane oxygenation (ECMO) are associated with a high incidence of complications (bleeding and tamponade requiring reexploration, right ventricular failure, respiratory failure, acute respiratory distress syndrome and pulmonary edema, neurologic complications, renal and hepatic failure, and infection) [5], and patients with complications are likely to require sedation and mechanical ventilation for a longer time period in ICU [52]. These patients could benefit from the sevoflurane effect over organs flow not only during the intraoperative, but also during the postoperative recovery period in the ICU.

### 4.4. Study Limitations

The present study is subject to a series of limitations. First, the LVAD is designed to be used in patients with heart failure; therefore, our results may not be directly applicable in clinical practice, because we used a healthy heart, as described elsewhere [53, 54]. This limitation should be addressed in an animal cardiogenic shock model. Second, since we studied the short-term effects of anesthetics (propofol and sevoflurane) in animals with an LVAD, the long-term effects of these drugs on organ blood flow warrant further investigation. Third, the effects of inhaled anesthetics [26–28, 55] and the intravenous anesthesia (propofol, opioids) [56, 57] may be dose-dependent. The concentration of sevoflurane we used represents approximately 1 minimum alveolar concentration, which is similar to the concentration used in other studies that show beneficial effects in a model of ischemia-reperfusion after thoracic-aortic occlusion in pigs [58].

We found that sevoflurane could be superior to propofol with respect to blood flow in the brain, liver, and heart tissue in a porcine model with LVAD. These findings may have significant clinical implications for anesthesiologists regarding the choice of sevoflurane in patients with an LVAD.

## Figures and Tables

**Table 1:** Markers of tissue injury and nitric oxide in both groups (propofol and sevoflurane) at baseline and 30 minutes after implantation of a left ventricular assist device.

	Propofol *n* = 5	Sevoflurane *n* = 5	*P* values
ALT (U/L)			
Baseline	29 ± 2	25 ± 2	0.221
PS 30′	29 ± 2	23 ± 2	0.048^*∗*^
AST (U/L)			
Baseline	50 ± 10	35 ± 3	0.116
PS 30′	94 ± 46	44 ± 3	0.358
Bilirubin (mg/dL)			
Baseline	0.25 ± 0.06	0.13 ± 0.02	0.081
PS 30′	0.24 ± 0.02	0.12 ± 0.04	0.028^*∗*^
GGT (U/L)			
Baseline	63 ± 12	55 ± 8	0.584
PS 30′	62 ± 22	47 ± 8	0.496
AP (U/L)			
Baseline	82 ± 8	72 ± 8	0.428
PS 30′	89 ± 12	79 ± 7	0.507
LDH (U/L)			
Baseline	330 ± 19	331 ± 13	0.943
PS 30′	374 ± 18	347 ± 27	0.420
Creatinine (mg/dL)			
Baseline	0.44 ± 0.03	0.57 ± 0.06	0.085
PS 30′	0.45 ± 0.03	0.47 ± 0.03	0.596
Urea (mg/dL)			
Baseline	27.2 ± 2.2	22.2 ± 0.9	0.059
PS 30′	28.2 ± 2.6	22.2 ± 1.2	0.053
Lactic acid			
Baseline	1.5 ± 0.5	1.1 ± 0.2	0.453
PS 30′	1.5 ± 0.3	1.2 ± 0.2	0.434
NO (*μ*M)			
Baseline	418 ± 47	691 ± 47	0.056
PS 30′	280 ± 92	478 ± 92	0.270

Data are expressed as the mean ± standard error of the mean. ALT: alanine transaminase; AST: aspartate aminotransferase; GGT: gamma-glutamyl transpeptidase; AP: alkaline phosphatase (AP); LDH: lactate dehydrogenase; NO: nitric oxide; PS: partial support. Statistically significant differences are shown. ^*∗*^ *P* < 0.05 propofol versus sevoflurane.

**Table 2:** Hemodynamic parameters in both groups (propofol and sevoflurane) at baseline and 30 minutes after implantation of a left ventricular assist device.

	Propofol *n* = 5	Sevoflurane *n* = 5	*P* values
HR (beats/min)			
Baseline	95 ± 4	89 ± 9	0.546
PS 30′	101 ± 6	101 ± 6	0.964
AP_m_ (mmHg)			
Baseline	70 ± 3	65 ± 5	0.384
PS 30′	65 ± 8	74 ± 7	0.404
PAP_m_ (mmHg)			
Baseline	23 ± 2	25 ± 2	0.506
PS 30′	27 ± 1	33 ± 3	0.083
CVP (mmHg)			
Baseline	15 ± 1	15 ± 1	0.856
PS 30′	14 ± 3	16 ± 2	0.584
CPP (mmHg)			
Baseline	18 ± 1	18 ± 1	0.471
PS 30′	15 ± 0.5	19 ± 1	0.052
SVRI			
Baseline	1583 ± 199	1368 ± 143	0.450
PS 30′	1128 ± 173	1433 ± 234	0.351
PVRI			
Baseline	171 ± 65	159 ± 32	0.877
PS 30′	217 ± 37	339 ± 85	0.269
CO (L/min)			
Baseline	2.4 ± 0.3	3 ± 0.3	0.185
PS 30′	2.5 ± 0.4	3.1 ± 0.4	0.347
SvO_2_ (%)			
Baseline	77 ± 4	82 ± 3	0.429
PS 30′	82 ± 1	89 ± 3	0.150
*T* (°C)			
Baseline	35.1 ± 0.2	35.9 ± 0.3	0.080
PS 30′	33.9 ± 0.4	34.6 ± 0.4	0.332

Data are expressed as the mean ± standard error of the mean. HR: heart rate; AP_m_: mean arterial blood pressure; PAP_m_: pulmonary artery mean pressure; CVP: central venous pressure; CPP: pulmonary capillary wedge pressure; SVRI: systemic vascular resistance index; PVRI: pulmonary vascular resistance index; CO: continuous cardiac output; SvO_2_: mixed venous oxygen saturation; *T*: temperature; PS: partial support.

**Table 3:** Hematologic parameters and blood gas analysis in both groups (propofol and sevoflurane) at baseline and 30 minutes after implantation of a left ventricular assist device.

	Propofol *n* = 5	Sevoflurane *n* = 5	*P* values
pH			
Baseline	7.4 ± 0.03	7.4 ± 0.02	0.314
PS 30′	7.3 ± 0.03	7.4 ± 0.02	0.583
PO_2_ (mmHg)			
Baseline	503 ± 24	425 ± 42	0.147
PS 30′	492 ± 43	483 ± 25	0.867
PCO_2_ (mmHg)			
Baseline	35 ± 2	38 ± 2	0.428
PS 30′	38 ± 3	42 ± 3	0.322
HCO_3_^−^ (mmol/L)			
Baseline	22 ± 1	26 ± 1	0.073
PS 30′	21 ± 1	24 ± 1	0.052
Hb (g/dL)			
Baseline	7.0 ± 0.1	7.4 ± 0.4	0.337
PS 30′	8.0 ± 0.5	8.3 ± 0.7	0.730
Hct (%)			
Baseline	19.7 ± 0.3	21.9 ± 1.2	0.148
PS 30′	22.5 ± 1.4	24.5 ± 2.0	0.452

Data are expressed as the mean ± standard error of the mean. PO_2_: partial pressure of oxygen; PCO_2_: partial pressure of carbon dioxide; HCO_3_^−^: bicarbonate; Hb: hemoglobin; Hcto: hematocrit; PS: partial support.
