# Corrigendum to “Effects of Sevoflurane and Propofol on Organ Blood Flow in Left Ventricular Assist Devices in Pigs”

**DOI:** 10.1155/2016/4152173

**Published:** 2016-08-17

**Authors:** Paloma Morillas-Sendín, Emilio Delgado-Baeza, María Jesús Delgado-Martos, Mónica Barranco, Juan Francisco Del Cañizo, Manuel Ruíz, Begoña Quintana-Villamandos

**Affiliations:** ^1^Department of Anesthesiology and Intensive Care, Gregorio Marañón University General Hospital, 28007 Madrid, Spain; ^2^Department of Experimental Medicine and Surgery, Gregorio Marañón University General Hospital, 28007 Madrid, Spain; ^3^Department of Cardiac Surgery, Gregorio Marañón University General Hospital, 28007 Madrid, Spain; ^4^Department of Pharmacology, Faculty of Medicine, Complutense University, 28040 Madrid, Spain

In the article titled “Effects of Sevoflurane and Propofol on Organ Blood Flow in Left Ventricular Assist Devices in Pigs” [1], there was an error in “Acknowledgments,” which should be corrected as follows.

This work was supported by FIS (PI08/1480, PI13/01261) and Fondos Feder.
